# Supplementary material for: Majority clustering for imbalanced image classification
Source: PeerJ Comput Sci. 2025 Jun 30;11:e2891. doi: 10.7717/peerj-cs.2891 (PMC12453654; doi:10.7717/peerj-cs.2891)
Supplement: Supplemental Information 6 [file peerj-cs-11-2891-s006.docx]

**Datasets:**

1. bald/no-bald: <https://www.kaggle.com/datasets/jessicali9530/celeba-dataset>
2. normal/tuberculosis: · <https://www.kaggle.com/datasets/roshanmaur/imbalanced-tuberculosis-and-pnuemonia-dataset>
3. covid/no-covid: [https://www.kaggle.com/datasets/roshanmaur/imbalanced-tuberculosis-and- pnuemonia-dataset](https://www.kaggle.com/datasets/roshanmaur/imbalanced-tuberculosis-and-%20pnuemonia-dataset)

**Computing infra:**

OS: Ubuntu 22.04.4 LTS

CPU: Intel(R) Core(TM) i5-6600

GPU: NVIDIA GeForce RTX 3090

RAM: 32 GB
