# Supplementary material for: Majority clustering for imbalanced image classification
Source: PeerJ Comput Sci. 2025 Jun 30;11:e2891. doi: 10.7717/peerj-cs.2891 (PMC12453654; doi:10.7717/peerj-cs.2891)
Supplement: Supplemental Information 7 [file peerj-cs-11-2891-s007.docx]

**Motivation behind the Proposed Technique**

The motivation behind this work stems from the pervasive issue of class imbalance in image classification tasks, where the distribution of classes is often skewed, leading to biased model performance. Traditional classification methods can struggle to accurately identify minority classes due to their limited representation in the dataset.

The proposed approach, MCIIC (Majority Clustering for Imbalanced Image Classification), aims to address this challenge by employing an under-sampling strategy that leverages unsupervised clustering techniques. By partitioning the majority class into distinct clusters, the method not only reduces the number of majority samples but also transforms the binary classification problem into a multi-class challenge. This transformation broadens the scope of classification and enhances model performance.

Utilizing the elbow method to determine the optimal number of clusters ensures that the clustering is both efficient and meaningful. By assigning new labels to these clusters, the method helps balance the representation of classes in the dataset, thereby reducing the deviation between majority and minority samples. This innovative approach seeks to improve the overall accuracy and reliability of image classification models, making them more robust in handling imbalanced datasets.
