# Supplementary material for: Majority clustering for imbalanced image classification
Source: PeerJ Comput Sci. 2025 Jun 30;11:e2891. doi: 10.7717/peerj-cs.2891 (PMC12453654; doi:10.7717/peerj-cs.2891)
Supplement: Supplemental Information 9 [file peerj-cs-11-2891-s009.docx]

**Description Of Model Used**

The proposed model consists of the following steps as shown in Fig. 1.

| Analyzing the Performance  9  Validating the Trained Model  9  Training the Multi-Class Data  9  Dividing the Majority Class (Multi-Class)  9  Input Dataset  Feature Extraction |
| --- |

**Figure 1**: Working Methodology

***Input Dataset***: The proposed model MCIIC is used to solve the problem of classification where the dataset is class imbalanced. The number of instances belonging to one class greatly out numbers the instances in another class. The dataset has binary classes, and the occurrence of one class is much less frequent than others. Several machine learning algorithms exhibit a bias towards the majority class, resulting in suboptimal predictive performance for the minority class. To deal with such problem, data need to be pre-processed before classifying. In MCIIC, the dataset used are of large size, so first stage involves extraction of features.

***Feature extraction***

Feature extraction plays important role for effectively handling large image datasets by reducing dimensionality, helps to enhance the generalization of the image. To perform this task, the model uses Residual Network (ResNet-18). The key advancement in ResNet is the introduction of Residual blocks. ResNet is a combination of traditional CNNs with residual blocks, where the residual blocks allow the network to extract more advanced and hierarchal features. These residual connections allow the model to propagate gradients effectively through deep layers, allowing the learning of complex features. The ResNet-18 model significantly decreases the complexity of the image by reducing its large dimensions to just 512 features. The ResNet model is pre-trained on an ImageNet dataset and can classify images into one thousand object categories. Fig. 2 shows the layered structure of a normal ResNet-18 model [10].


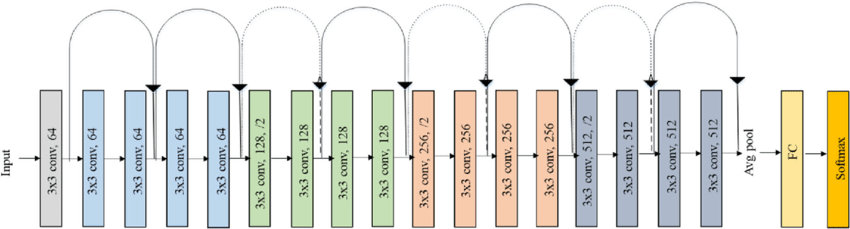


**Figure 2:** Architecture of ResNet-18 Model

In the proposed model a ResNet-18 model (originally trained on ImageNet data set) is used as base. The ResNet-18 model comprises of eighteen layers, where the first 17 layers are used for feature extraction and final layer is responsible for predicting the image label. These initial layers are crucial for extracting significant features (512 features) from the input images. These extracted features are stored in an array. Subsequently, K-means clustering is used to divide majority class into multiple classes based on the extracted features.

***Elbow method***

In the proposed model, to address class imbalance in the data, the majority class is partitioned into multiple clusters using k-means clustering. The optimal number of clusters (k) is determined using the elbow method. This approach requires minimizing a sum of squared errors (SSE):

$SSE=\sum_{k=1}^{cluster\_n} \sum_{x_{j}\in X} \left\| x_{i}-c_{k} \right\|$ (1)

where k =N_c_ is the optimal number of clusters where the elbow is formed in the graph, x_i_ is pixel value of an image in the dataset and c_k_ is the cluster centre which is initialized manually and cluster_n is the maximum number of clusters to be considered.

| **Input:**  - Data: Majority samples for clustering  - cluster_n: Maximum number of clusters to consider  **Output:**  - Optimal number of clusters (k)  1. Initialize an empty list to store the SSE values for different values of k.  2. For each value of k from 1 to cluster_n:  a. Perform k-means clustering on the data, using k clusters.  b. Calculate the total within-cluster sum of squared error (SSE) for the clustering.  c. Append the SSE value to the list.  3. Plot a curve between the values of k (x-axis) and the corresponding SSE values (y-axis).  4. Identify the "elbow point" on the plot, where the rate of decrease in SSE slows down significantly.  5. The value of k at the elbow point is assumed as the optimal number of clusters.  6. Return the optimal number of clusters (k). |
| --- |

**Algorithm for elbow method**

Fig. 3 shows the graph of elbow method for covid/no-covid test dataset providing the optimal value of k as the output. The below given graph forms a curve at k=5. The obtained value of k is the number of classes in which the majority class will be divided using k-means clustering.


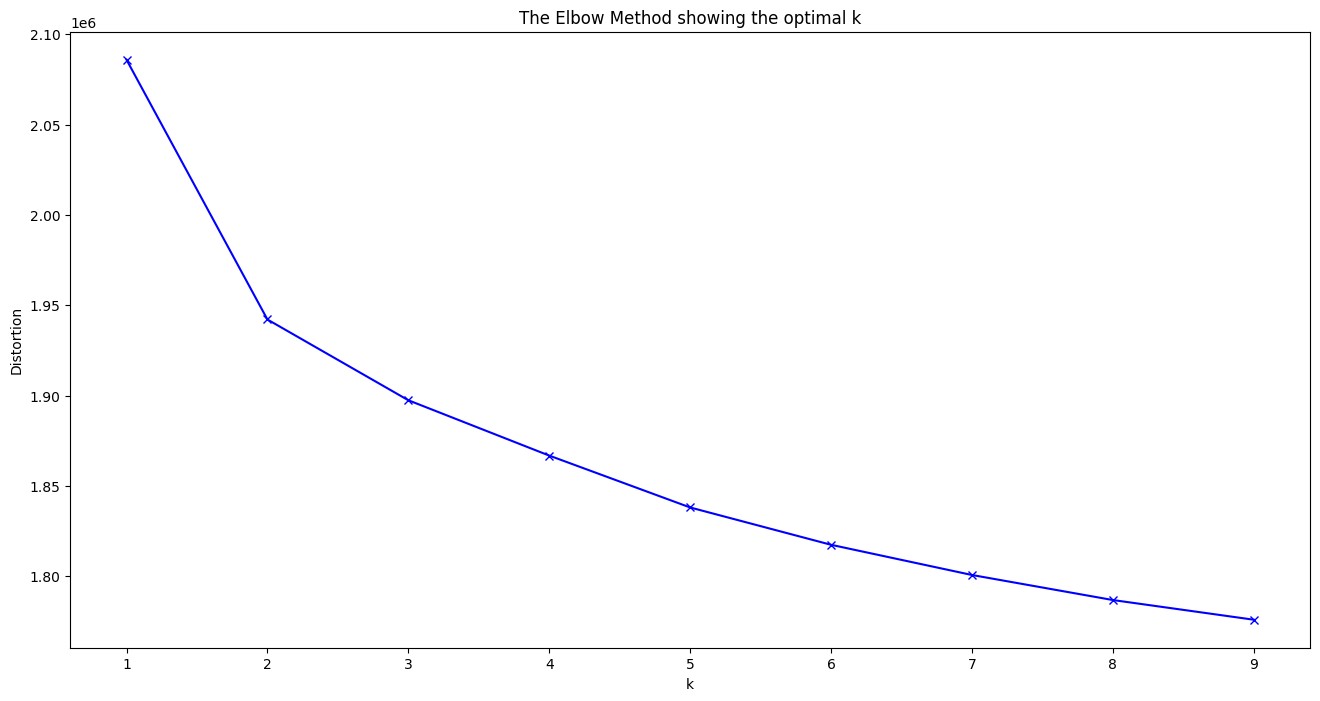


**Figure 3:** Graph of Elbow method for covid/No-covid dataset

According to the elbow method, the determined number of clusters is applied to partition the majority class. The dataset M1 representing the majority class is segmented into Nc clusters using k-means clustering, with each cluster assigned a new class label. This procedure results in a reduction of the number of images per class as the number of majority classes increases to N_c_. Consequently, this transforms the original binary-class problem into a multi-class problem with N_c_+1 total classes, where the (N_c_+1)^th^  class represents the minority class.

***Training the model using Transfer Learning***

Once the data is classified into N_c_+1 classes, it can be used to train the required model. These models can be trained from scratch i.e. training a neural network without relying on pre-existing knowledge or pre-trained weights. Alternatively, there is an option of employing machine learning techniques such as transfer learning to train new models based on previously trained models, particularly useful when dealing with smaller datasets.

Transfer learning is a concept of machine learning which involves using a pretrained neural network model to improve the learning process on another task, i.e. model trained on one task is adapted for second task [8]. The process of transfer learning involves selecting a pretrained model (ResNet-18 model, trained on a large data set), removing the ending layers responsible for specific tasks, and finally introducing new layers after the original layers. These layers are responsible for training the model for new tasks. The last step involves training the model for the new task using new data sets.

Transfer learning is often considered better than training a model from scratch because it provides data efficiency, faster convergence, and improved generalization. In many real-world scenarios, obtaining a large labelled dataset for a specific task can be challenging and expensive. By leveraging a pre-trained model's learned features from a related task, transfer learning enables the adaptation to a target task even with limited labelled data. As the pre-trained model has already learnt generic features from a different but related task, transfer learning provides quicker convergence and reduces resource requirements. Fig. 4 shows the difference between training a model from scratch vs transfer learning as learned knowledge is transferred while training the new model resulting into improved efficiency [9].

Thus, the goal of using transfer learning for training the model is to leverage a pretrained model’s features without extensively retraining the complete model. Dataset is split into 80-10-10 ratio for training, validation and testing. Training is now performed on the new multiclass dataset using transfer learning. Resnet-18 pretrained on ImageNet dataset, is used for training the dataset to achieve the desired outcomes.

| 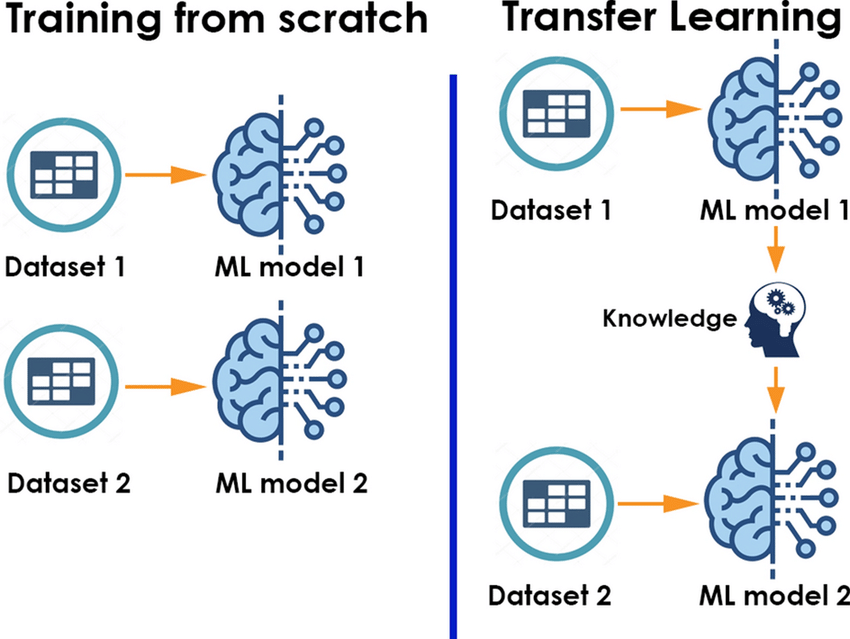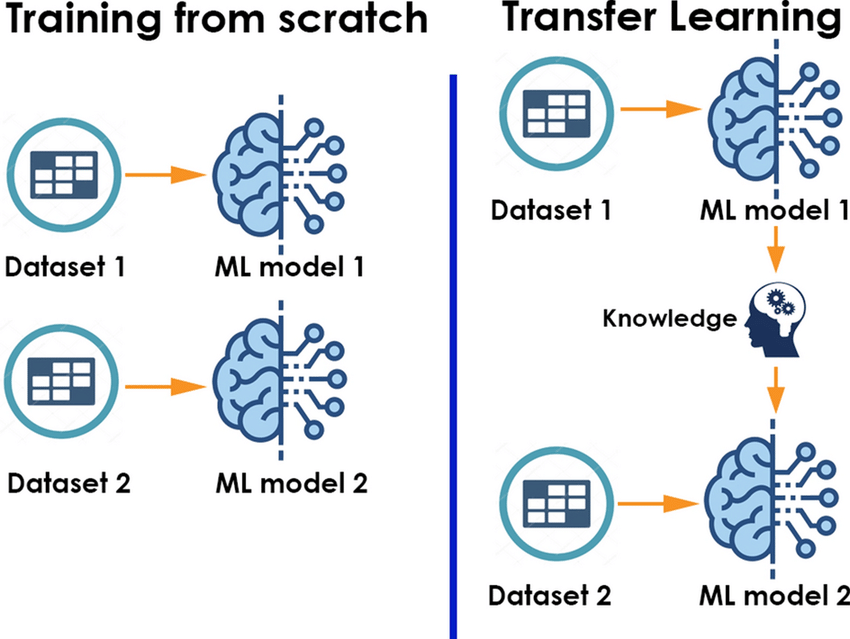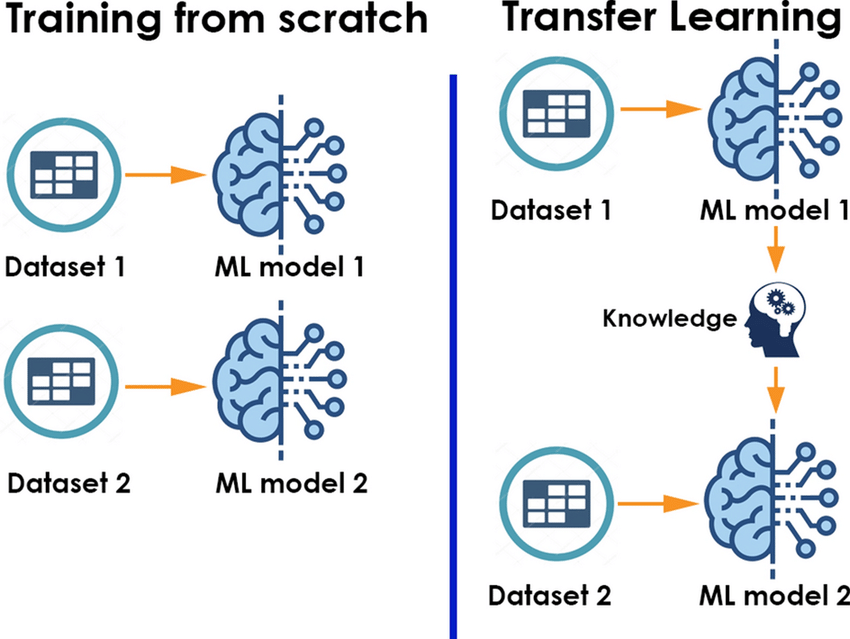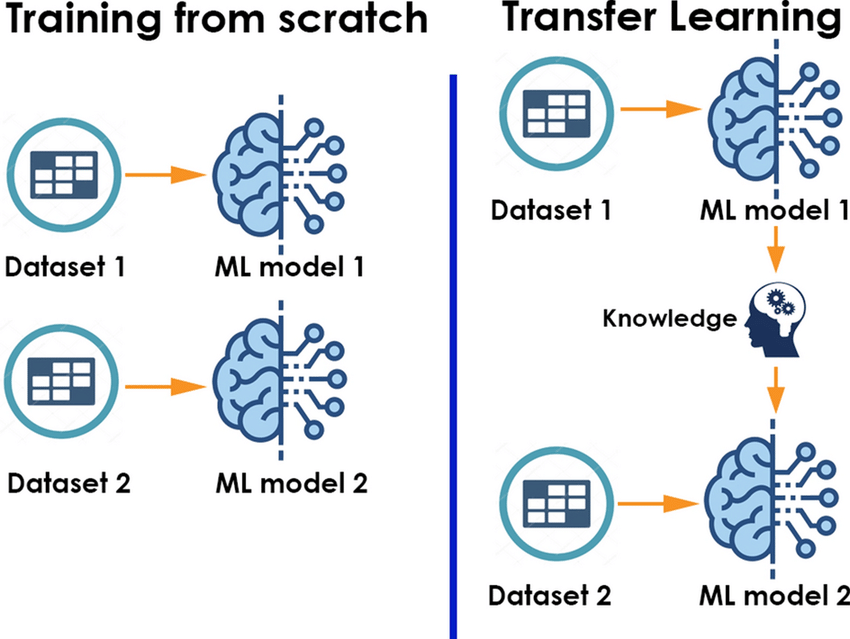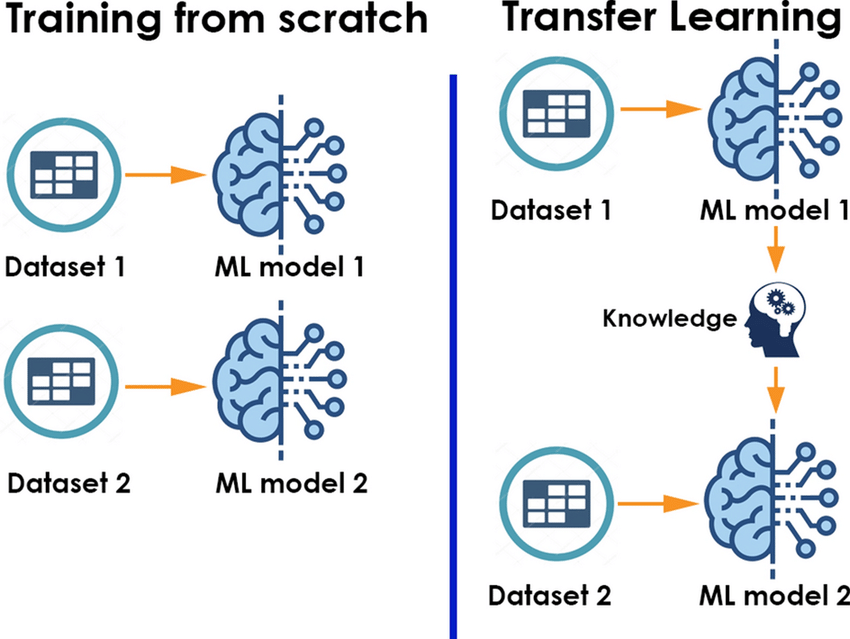  (b)  (a)  Transfer of Knowledge  Dataset 1  Dataset 2  **ML Model 2**  **ML Model 1**  Dataset 2  Dataset 1  **ML Model 2**  **ML Model 1** |
| --- |

**Figure 4**: (a) Training model from scratch (b) Training the model using Transfer learning

***Validating the Training Model and Tuning of the Hyper-Parameters***

During the training process, the model's weights are continuously updated to minimize a chosen loss function (e.g., cross-entropy loss) on the training data. However, to prevent overfitting and ensure the model generalizes well to unseen data, the model's performance on the validation set is monitored. The best model weights are those that result in the highest accuracy (or lowest loss) on the validation set.
