# Supplementary material for: Majority clustering for imbalanced image classification
Source: PeerJ Comput Sci. 2025 Jun 30;11:e2891. doi: 10.7717/peerj-cs.2891 (PMC12453654; doi:10.7717/peerj-cs.2891)
Supplement: Supplemental Information 10 [file peerj-cs-11-2891-s010.docx]

**Limitation of The Proposed Methodology**

While the MCIIC (Majority Clustering for Imbalanced Image Classification) approach presents a novel solution to the class imbalance problem, it has several limitations:

1. **Clustering Sensitivity**: The effectiveness of the proposed method heavily relies on the clustering algorithm used and the parameters set (e.g., the choice of distance metric). Inappropriate clustering can lead to suboptimal partitions and misrepresentations of the majority class.
2. **Class Overlap**: Clusters formed from the majority class may overlap, which can complicate the classification process and lead to mislabeling of samples, particularly when the minority class samples are similar to majority class samples.
3. **Assumption of Cluster Homogeneity**: The method assumes that the majority class can be effectively partitioned into homogeneous clusters. If the class distribution is highly heterogeneous, this assumption may not hold true.
4. **Limited Generalization**: The performance of the MCIIC method may vary across different datasets and applications. Its effectiveness may not generalize well to all scenarios, particularly those with highly diverse or complex class structures.
5. **Dependence on Labeling**: Assigning new labels to clusters requires careful consideration and may introduce bias, particularly if the labels do not accurately represent the underlying data distribution.

By acknowledging these limitations, future research can focus on refining the MCIIC method or exploring complementary techniques to enhance its robustness and applicability in various classification tasks.
